# C3 Opsonization of Anthrax Bacterium and Peptidoglycan Supports Recognition and Activation of Neutrophils

**DOI:** 10.3390/microorganisms8071039

**Published:** 2020-07-13

**Authors:** Narcis I. Popescu, Ravi S. Keshari, Jackie Cochran, K. Mark Coggeshall, Florea Lupu

**Affiliations:** 1 Department of Arthritis and Clinical Immunology, Oklahoma Medical Research Foundation, Oklahoma City, OK 73104, USA; jackie-cochran@omrf.org (J.C.); mark-coggeshall@omrf.org (K.M.C.); 2 Cardiovascular Biology Research Program, Oklahoma Medical Research Foundation, Oklahoma City, OK 73104, USA; Ravi-Keshari@omrf.org

**Keywords:** neutrophils, *Bacillus anthracis*, anthrax, peptidoglycan, complement, C3

## Abstract

Neutrophils are the most abundant innate cell population and a key immune player against invading pathogens. Neutrophils can kill both bacterium and spores of *Bacillus anthracis*, the causative anthrax pathogen. Unlike interactions with professional phagocytes, the molecular recognition of anthrax by neutrophils is largely unknown. In this study, we investigated the role of complement C3 deposition on anthrax particles for neutrophil recognition of bacterium and/or its cell wall peptidoglycan, an abundant pathogen-associated molecular pattern that supports anthrax sepsis. C3 opsonization and recognition by complement receptors accounted for 70–80% of the affinity interactions between neutrophils and anthrax particles at subphysiologic temperatures. In contrast, C3 supported up to 50% of the anthrax particle ingestion under thermophysiologic conditions. Opsonin-dependent low affinity interactions and, to a lower extent, opsonin-independent mechanisms, provide alternative entry routes. Similarly, C3 supported 58% of peptidoglycan-induced degranulation and, to a lower extent, 23% of bacterium-induced degranulation. Interestingly, an opsonin independent mechanism mediated by complement C5, likely through C5a anaphylatoxin, primes azurophilic granules in response to anthrax particles. Overall, we show that C3 deposition supports anthrax recognition by neutrophils but is dispensable for pathogen ingestion and neutrophil degranulation, highlighting immune recognition redundancies that minimize the risk of pathogen evasion.

## 1. Introduction

*Bacillus anthracis* spores cause anthrax infections upon penetration of the respiratory, cutaneous or gastrointestinal barriers [1,2]. Primarily a zoonotic disease with rare natural occurrence in humans [3], ecologic niche modelling predicts an expansion of anthrax risk to both livestock and humans with continued climate changes [4]. In addition, the weaponization potential of anthrax spores [5] increases the risk for accidental [6] or intentional [7] release of the pathogen in localized populations. Regardless of vectors of colonization, left unchecked, anthrax infections can progress to fulminant disease characterized by systemic, hematogenous, pathogen dissemination, bacteremia, sepsis and multiple organ failure [8], implying the pathogen can overcome host defense mechanisms.

Innate immune defenses are critical for the seeding and progression of anthrax, and may explain differences in infection kinetics observed with inhalational and cutaneous disease. During inhalational anthrax, spores are rapidly internalized by professional phagocytes at the pulmonary level [9], a process enhanced by the complement C3b deposition [10,11] and complement receptors (CRs) [12]. The pathogen escapes phagolysosomal processing [13] either at primary sites or during relocation to the mediastinal lymph nodes [14]. Subsequent bacterial growth and secretion of virulence factors, such as the tripartite exotoxins [15] and the poly-γ-d-glutamate capsule [16], support immune evasion and contribute to a rapid progression to late stage disease. Cutaneous anthrax, however, is usually localized to sites of infection and rarely evolves to systemic disease [17]. Neutrophils are the primary innate immune cells recruited at sites of cutaneous infection, and have been proposed to limit anthrax dissemination [18]. In contrast, earlier inhalational anthrax studies recognized only a minor contribution from neutrophils to disease progression [19]. Furthermore, newer reports highlight the role of neutrophils during systemic disease [20], which may require priming through the inflammasome/IL-1β axis [21]. Similarly, we previously reported rapid activation of neutrophils in a non-human primate model of late stage systemic anthrax [22], but specific neutrophil contributions to septic pathophysiology were harder to isolate in this model.

Neutrophils can efficiently kill both anthrax spores and vegetative bacteria, either through oxidative stress inside phagolysosomes and/or the secretion of antimicrobial peptides [20,23]. They can be recruited at sites of infection [18,24,25], or we can encounter the pathogen during the hematogenous dissemination to secondary organs. Neutrophil-mediated bacterial killing, either phagocytosis-dependent and/or mediated by degranulation, was reportedly insensitive to the presence of classical virulence factors, the antiphagocytic capsule and exotoxins [20,23]. It is worth noting, nevertheless, that toxin paralysis of neutrophil functions has been reported in other instances [26,27,28], and could reflect different states of neutrophil activation. Taken together, these studies raise the possibility that neutrophils, one of the primary defenses against infectious pathogens, could employ different strategies for the recognition of anthrax as opposed to antigen-presenting phagocytes. Yet, very little is known about the mechanisms governing the neutrophil recognition of anthrax bacterium.

Innate immune cells, including neutrophils, recognize pathogens through a heterogeneous array of innate immune receptors, such as immunoglobulin [29], complement [30,31] and toll-like receptors [32], to name a few. They provide overlapping and sometimes redundant signals that promote specific phenotypic outcomes in effector cells. Given that the complement C3b deposition is critical for the recognition of anthrax particles by professional phagocytes [10,11,12], we thought to investigate and define the role played by complement opsonization in anthrax recognition by, and with the activation of, naïve human neutrophils. In parallel, we investigated neutrophil recognition of anthrax peptidoglycan (PGN), the main constituent of the cell wall of Gram-positive bacteria, which we [33], and others [34], have shown to contribute to late-stage anthrax pathology. We found that the C3b deposition on bacteria and/or peptidoglycan is required for affinity receptor recognition at subphysiologic temperatures, but it is dispensable at 37 °C, which is where other low affinity interactions could come in play. While C3b recognition contributes to about half of the serum-dependent internalization of anthrax particles, redundant mechanisms, both dependent and independent of serum opsonins, are in play. Similarly, C3b recognition by complement receptors supports, but is not essential for, the degranulation of azurophilic granules, which is aided by a priming event mediated by complement C5.

## 2. Materials and Methods

### 2.1. Materials

Blood leukocytes separation media Histopaque-1077 was from Sigma (St. Louis, MO, USA). Cell culture RPMI-1640 media and supplements were from ATCC (Manassas, VA, USA). Cell culture grade Hank’s buffer salt solution without calcium or magnesium (HBSS) was from Sigma, and the purified bovine serum albumin (BSA), magnetic-activated cell sorting grade, was from Miltenyi Biotec (Auburn, CA, USA). *Bacillus anthracis*, Strain Sterne BA781 (Δ*lef243*/Δ*cya244*/Δ*pagA242*), was obtained through BEI Resources (NR-9401), National Institute of Allergy and Infectious Diseases, National Institutes of Health. Trypticase Soy Broth was from BD Biosciences (San Jose, CA, USA). Bacteria and PGN labelling reagents included FITC (fluorescein isothiocyanate, Sigma, St. Louis, MO, USA) and pHrodo iFL Red STP Ester (Life Technologies, Carlsbad, CA, USA). The C3 convertase complement inhibitor compstatin was from Tocris Bioscience (Minneapolis, MN, USA). All complement factor immunodepleted sera were obtained from Complement Technology Inc. (Tyler, TX, USA), and the manufacturer confirmed the specificity of immunodepletion with no off-target reductions in other complement factors. The phorbol-12-myristate-13-acetate (PMA) used as a positive control agonist in select experiments was from EMD Millipore (Billerica, MA, USA). The myeloperoxidase substrate ADHP (10-acetyl-3,7-dihydroxyphenoxazine) was obtained from AAT Bioquest (Sunnyvale, CA, USA). The flow cytometry monoclonal antibodies used in this study included allophycocyanin (APC) labelled anti-human CD16 (clone 3G8, BD Biosciences, San Jose, CA, USA), and FITC-labelled anti-human MPO antibodies (clone MPO455-8E6, eBioscience, San Diego, CA, USA). APC or FITC labelled IgG1 kappa isotype controls were from eBioscience, San Diego, CA, USA (clone P3.6.2.8.1). All chemicals used to prepare buffers in house were ACS grade or better and were obtained from Sigma Aldrich (St. Louis, MO, USA) or Fisher Scientific (Waltham, MA, USA).

### 2.2. Analysis of Primary Human Neutrophils

Studies on peripheral blood leukocytes were conducted in accordance with the Declaration of Helsinki, and the protocol was approved by the Institutional Review Board at the Oklahoma Medical Research Foundation (protocol number 19-11 approved on 13 March, 2019). Voluntary participants were informed of study aims and procedures, and gave written informed consent. Samples were de-identified and coded by the study phlebotomist before experiments. Peripheral blood leukocytes were separated by density gradient centrifugation using Histopaque-1077, the PBMC layer was discarded and polymorphonuclear leukocytes (PMNs) were pooled and subjected to two rounds of hypotonic ACK lysis (155 mM NH_4_Cl, 10 mM KHCO_3_, 0.1 mM EDTA). PMNs were washed twice with HBSS containing 1% BSA (HBSS-BSA), and then were counted and kept in HBSS-BSA or transferred to RPMI-1640 media supplemented with glucose and glutamine. Unless otherwise noted, neutrophils were stimulated at 37 °C in a humidified atmosphere containing 5% CO_2_.

### 2.3. Bacteria Strain and Peptidoglycan Purification

*Bacillus anthracis* Strain Sterne BA781 (Δ*lef243*/Δ*cya244*/Δ*pagA242*) was grown in Trypticase Soy Broth media and monitored spectrophotometrically until the optical density at 600 nm reached 0.8. Bacteria was pelleted by centrifugation, washed twice with HBSS and resuspended in 1/10 volumes HBSS. An aliquot was used to quantify titer by plating serial dilutions of bacterial suspension, and the remaining suspension was heat inactivated for 1 h at 70 °C in a water bath. Inactivation was confirmed by plating. Heat inactivated bacteria (hk*Ba*) was aliquoted and stored frozen (−80 °C) until use. In all assays, hk*Ba* was used at 1 × 10^7^ cfu equivalents/mL, a concentration eliciting proinflammatory and procoagulant responses similar to 20 µg/mL PGN in previous studies [35].

PGN was purified from parental bacteria (Sterne Strain BA781) as reported [36,37]. Quality control analysis is described in detail elsewhere [37,38]; purified PGN preparation was free of TLR2 and TLR4 agonists [37], and promoted proinflammatory and procoagulant responses in primary human monocytes [35,39].

### 2.4. Neutrophil Recognition of hkBa and PGN

Bacteria (hk*Ba*) and PGN were labelled with FITC according to manufacturer’s protocols (Sigma, St. Louis, MO, USA). PMNs were purified on the day of the experiment, transferred into RPMI-1640 and chilled on ice for 20 min to minimize internalization. Where needed, compstatin was added to autologous human serum (AHS) at 0.2 mg/mL for 20 min before particle opsonization. FITC-labelled hk*Ba* or PGN were preopsonized for 1 h at 37 °C with AHS with or without compstatin, or with C1q-, C3- or C5-immunodepleted sera, all from Complement Technology Inc. (Tyler, TX, USA). Preopsonized particles were chilled on ice for at least 30 min, and then were added to PMN suspensions. Reactions were incubated for 30 min at room temperature, excess particles were washed and PMNs were fixed and immunostained on ice with allophycocyanin (APC) labelled anti-human CD16 (clone 3G8, BD Biosciences, San Jose, CA, USA). Binding and uptake of bacteria and PGN was quantified by flow cytometry after gating on neutrophils (CD16^high^ leukocytes). Data were collected on LSR II or FACSCelesta cytometers (BD Biosciences, San Jose, CA, USA). Histogram overlays are exemplifying a median responsive individual, while mean ± standard deviation (SD) is depicted for the study group (*n* = 4–8 independent donors).

### 2.5. Neutrophil Phagocytosis of hkBa and PGN

Bacteria (hk*Ba*) and PGN were labelled with the pH-sensitive, amine-reactive, pHrodo iFL Red STP Ester according to the manufacturer’s protocol (Life Technologies, Carlsbad, CA, USA). On the day of the experiment, pHrodo-labelled hk*Ba* and/or PGN were preopsonized for 1 h at 37 °C with autologous serum with or without compstatin (0.2 mg/mL), or with C1q-, C3- or C5-immunodepleted sera; then they were chilled on ice for at least 30 min. Freshly purified PMNs were counted and 2 × 10^5^ PMNs per reaction, were transferred into the wells of a black 96-well microplate (Sigma, St. Louis, MO, USA) and were chilled on ice for 30 min to minimize internalization. After pHrodo-hk*Ba* or PGN addition to ice, cells were transferred to a humidified 5% CO2 atmosphere at 37 °C for 1 h to phagocytose bacterial particles, and then were washed twice with ice-cold HBSS + 0.3% BSA, resuspended in 100 µL ice-cold HBSS-BSA and kept on ice until fluorescence quantitation to minimize further internalization. Plates were read on a Fluostar Omega microplate reader (BMG Labtech, Cary, NC, USA) using the 544 nm excitation and 590 nm emission filter set. An internal calibrator was added on each plate (either pHrodo-hk*Ba* or pHrodo-PGN diluted in phosphate-citrate buffer, pH 4.5) and was used to equivalate fluorescence readings at different acquisition gains. Average responses are depicted as mean ± SD for six independent donors, with each donor read in 2–4 replicates.

### 2.6. Neutrophil Degranulation

Neutrophil degranulation was assessed through quantitation of myeloperoxidase (MPO) released into supernatants by stimulated PMNs. Unlabeled hk*Ba* and/or PGN were preopsonized for 1 h at 37 °C with autologous serum with or without compstatin (0.2 mg/mL), or with C1q-, C3- or C5-immunodepleted sera. Purified neutrophils, 5 × 10^5^ PMNs per reaction, were added and reactions were further incubated for 2 h at 37 °C in a humidified 5% CO2 atmosphere. Samples were centrifuged at 500× *g* for 10 min at 4 °C, and supernatants were stored at −80 °C until MPO assessment.

MPO activity assays were performed essentially as described [40]. Briefly, conditioned supernatants were diluted 1:10 with PBS and loaded on a black 96-well microplate. An equal volume of MPO substrate (200 µM ADHP) containing 0.0003% H_2_O_2_ (hydrogen peroxide) was added to each well. Reactions were incubated for 15 min protected from light, and then were read on a Fluostar Omega microplate reader (BMG Labtech, Cary, NC, USA) using the 544 nm excitation and 590 nm emission filter set. MPO activity in unknown samples was extrapolated from standard curves of known activities obtained with purified human MPO. Data are depicted as mean ± SD for 10 independent donors assessed in duplicate.

### 2.7. MPO Immunoreactivity in Neutrophils

MPO immunoreactivity in activated neutrophils was quantified by flow cytometry using a FITC-labelled anti-human MPO antibody and standard intracellular staining protocols. Briefly, purified PMNs were transferred into RPMI-1640, and 1 × 10^6^ PMNs per reaction were incubated with preopsonized hk*Ba* or PGN as above. PMNs were stimulated for 2 h at 37 °C in a humidified 5% CO2 atmosphere to match degranulation conditions. Activated PMNs were washed, fixed with 2% paraformaldehyde and Fc receptors were blocked with 10% human AB serum (Sigma, St. Louis, MO, USA). PMNs were permeabilized with cell staining media (0.1% saponin, 5% newborn calf serum in PBS) and stained with FITC-labelled anti-human MPO (clone MPO455-8E6, eBioscience, San Diego, CA, USA) and APC-labelled anti-human CD16 (clone 3G8, BD Biosciences, San Jose, CA, USA). Data were acquired on LSRII or FACSCelesta cytometers (BD Biosciences, San Jose, CA, USA), and geometric means of MPO fluorescence intensity in CD16^high^ gated neutrophils were used for analysis. Acquisition thresholds were set using concentration matched isotype controls (clone P3.6.2.8.1, eBioscience, San Diego, CA, USA) run in parallel for each donor. Data are depicted as mean ± SD for 10 independent donors.

### 2.8. Data Analysis and Representation

Flow cytometry analysis, gating and histogram overlays were performed in FlowJo (v. 10.6.2, TreeStar Inc., Ashland, OR, USA). Thresholds were set using FMO (fluorescence minus one) controls, for bacteria and PGN binding, or isotype immunoglobulin staining run in parallel on the same donor. Data analysis was performed using Prism (v. 8.4.2, GraphPad Software, San Diego, CA, USA). Differences between two groups were analyzed by ratio paired *t* tests, while multiple groups were analyzed by repeated measures (RM) analysis of variance (ANOVA, San Francisco, CA, USA) with Holm–Sidak’s multiple comparisons tests. After pairwise normalization, we employed one sample *t* test to assess significant deviations from the reference value of 100 set by the normalization process. The threshold for statistical significance was set at *p* < 0.05, and adjusted *p*-values are conventionally represented across graphs (* *p* < 0.05, ** *p* < 0.01, *** *p* < 0.001, **** *p* < 0.0001). Individual panels were generated with Prism, and figures were collated in Adobe Illustrator (Adobe Inc., San Jose, CA, USA).

## 3. Results

### 3.1. Complement-Dependent Recognition of Anthrax Bacterium and Peptidoglycan

Complement opsonization of anthrax bacteria and spores have been shown to mediate pathogen internalization by professional phagocytes such as macrophages and dendritic cells [12,14], and this predominantly centers around the recognition of C3b/iC3b decorating the pathogen surface [10]. Although neutrophils are important mediators of innate immunity and efficiently kill both vegetative bacteria as well as anthrax spores [20,23], the molecular recognition of anthrax by neutrophils has not been detailed. We thus examined whether the deposition of complement opsonins aid in the neutrophil recognition of anthrax bacterium and its cell wall PGN, a major pathogen associated molecular pattern (PAMP) that supports the pathology of late stage anthrax sepsis [33]. We centered our approach on reducing the availability of C3 opsonin using either compstatin, a tridecapeptide inhibitor that binds C3 and prevents its proteolytic activation by the C3 convertase [41], or the specific immunodepletion of C3. In addition, C1q depletion informs about the potential involvement of the upstream opsonin C1q being recognized by multiple complement receptors, while also reducing C3 deposition through the classical complement pathway. In contrast, C5 depletion is expected to abolish formation of the terminal complement complex without reducing the deposition of upstream opsonins.

**Figure 1:**
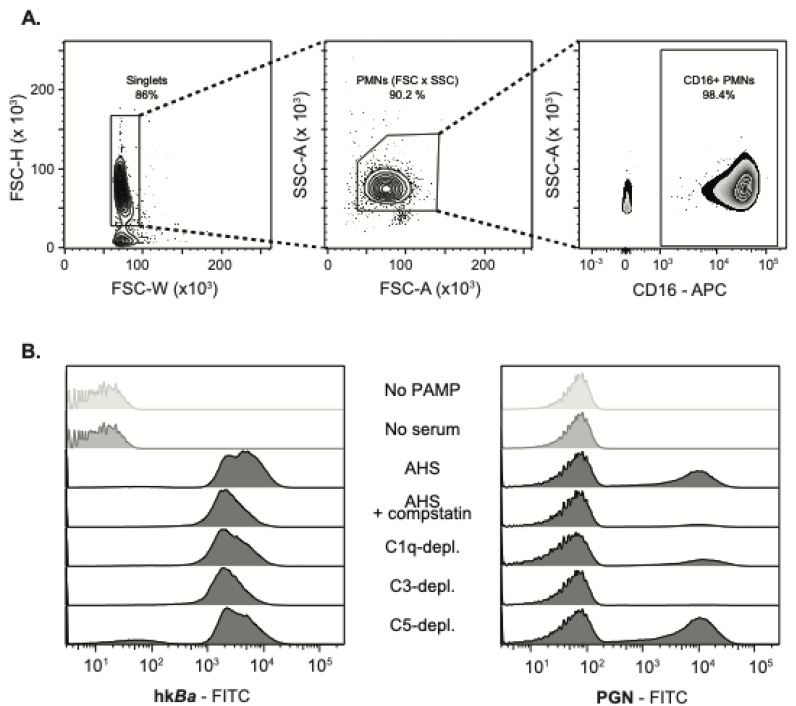
The interaction between polymorphonuclear neutrophils (PMNs) and *Bacillus anthracis* (hk*Ba*) or anthrax peptidoglycan (PGN) is dependent on the complement opsonization of pathogen particles. (**A**) Flow cytometry gating strategy and exemplification of neutrophil purification (CD16^high^ leukocytes). (**B**) Histogram overlay of FITC-labelled hk*Ba* (left) and/or PGN (right) interaction with CD16^high^ PMNs isolated from a median responsive individual, in the presence of compstatin, a C3 convertase inhibitor, or the immunodepletion of complement factors.

**Figure 2:**
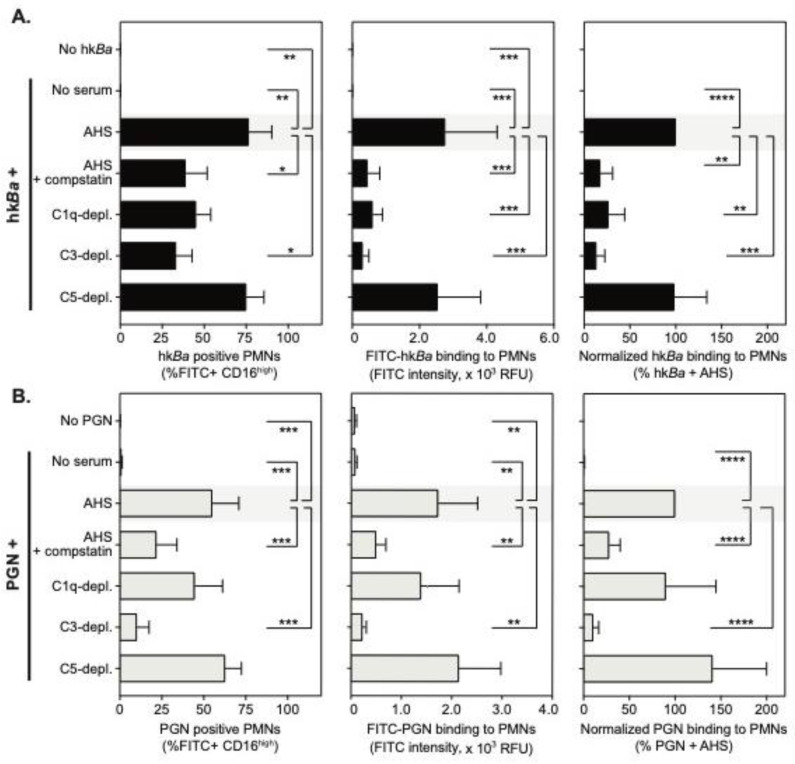
Flow cytometry quantitation of FITC-hk*Ba* (**A**) and/or the FITC-PGN (**B**) interaction with CD16^high^ neutrophils in the presence of compstatin, a C3 convertase inhibitor, or the immunodepletion of complement factors. Data, shown as mean ± SD of 4–8 independent donors, depict bacteria or PGN positive PMNs (left panels), total bacteria or PGN uptake (geometric mean of fluorescence intensity, middle panels) and normalized changes in fluorescence intensity compared to bacteria or PGN uptake in the presence of autologous serum (AHS) that is considered 100% (right panels). For each panel, statistically significant differences compared to PAMPs + AHS (shaded) are depicted graphically (* *p* < 0.05; ** *p* < 0.01; *** *p* < 0.001; **** *p* < 0.0001) and were computed either by repeated measures one-way analysis of variance (ANOVA) with Holm–Sidak’s multiple comparisons test (left and middle panels), or by one sample *t* test compared to the normalized value (right panel).

Human neutrophils were isolated by density gradient centrifugation and further identified by the elevated expression of FcγRIIIb recognized by the 3G8 anti-CD16 monoclonal (Figure 1A). At sub physiologic temperatures, neutrophils did not bind either FITC-labelled hk*Ba* or PGN (Figure 1B and Figure 2) in the absence of serum opsonization. Upon opsonization with the autologous serum, FITC labelled hk*Ba* associated with 76 ± 14% CD16^high^ PMNs, and anthrax PGN was similarly detected in 55 ± 16% neutrophils. These data confirm that affinity recognition of anthrax bacterium and PGN requires opsonization by serum factors.

Compstatin inhibition of C3b opsonization significantly reduced the recognition of both hk*Ba* and PGN (Figure 1B and Figure 2). After pairwise normalization of individual responses in the study cohort, compstatin reduced neutrophil associated hk*Ba* by 82 ± 14% (*p* = 0.0012, one sample *t* test compared to hk*Ba* binding in the presence of autologous serum), and similarly reduced PGN binding by 72 ± 12% (*p* = 0.0012 compared to PGN binding in the presence of autologous serum). C3 immunodepletion had a similar effect, reducing hk*Ba* recognition by 87 ± 9.2% (*p* = 0.0003) and PGN binding by 90 ± 6.1% (*p* < 0.0001). C1q immunodepletion reduced hk*Ba* recognition by 73 ± 18% (*p* = 0.0036) but did not significantly alter PGN recognition (10% mean reduction, *p* = 0.6181), which might reflect the preferential activation of the classical complement pathway on the bacterial surface. In contrast, anthrax PGN initiates complement activation through both the classical and lectin pathways [42]. As expected, upstream opsonin deposition was unhindered by C5 depletion, which did not inhibit recognition of either hk*Ba* or PGN (Figure 2). Taken together, our data indicate that affinity recognition of anthrax bacterium and PGN requires C3b opsonization of pathogen surfaces. Similar results were obtained in a subset of binding experiments performed on ice.

### 3.2. C3b Opsonization Requirements for the Internalization of Anthrax Bacterium and Peptidoglycan

**Figure 3:**
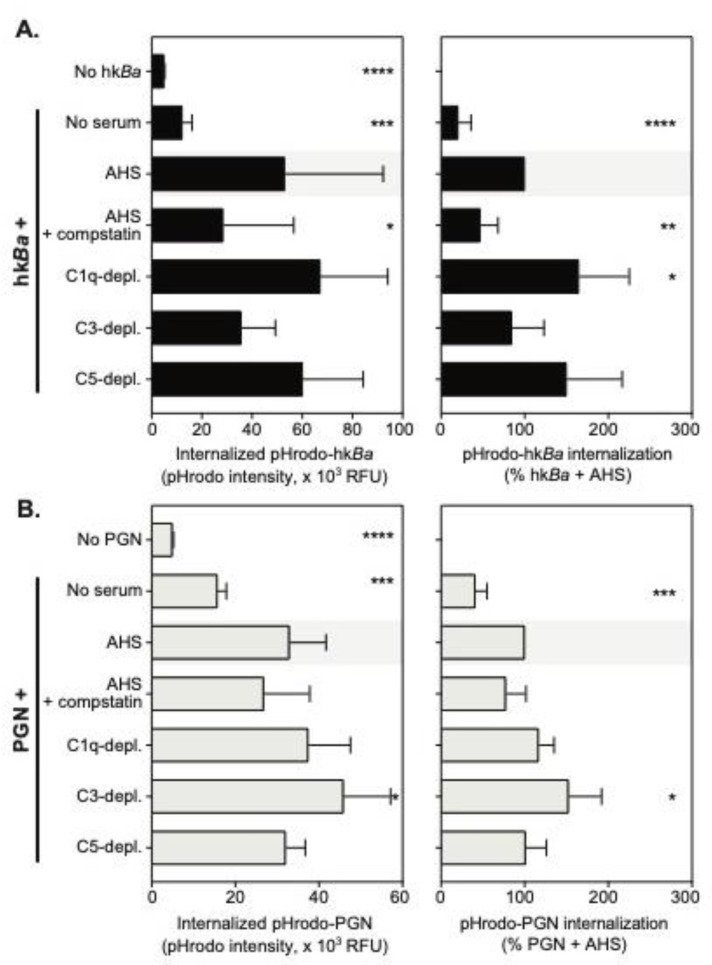
Quantitation of pHrodo-hk*Ba* (**A**) and/or pHrodo-PGN (**B**) internalization by neutrophils in the presence of compstatin, a C3 convertase inhibitor, or the immunodepletion of complement factors. Data are shown as mean ± SD of 6 independent donors, and depict pHrodo fluorescence intensity after the internalization of labeled bacteria or PGN (left panels) and normalized changes in endocytosed pHrodo-labeled particles compared to bacteria or PGN uptake in the presence of autologous serum (AHS), which was considered 100% (right panels). Statistically significant differences compared to pHodo uptake in the autologous serum (AHS, shaded) are depicted graphically (* *p* < 0.05; ** *p* < 0.01; *** *p* < 0.001; **** *p* < 0.0001), and were computed by repeated measures one-way ANOVA with Holm–Sidak’s multiple comparisons test (left panels), or one sample *t* test compared to the normalized value (right panels).

Recognition of complement opsonized particles supports neutrophil phagocytosis, oxidative burst and degranulation. Previous studies reported both phagolysosomal killing and the extracellular release of antimicrobial peptides [20,23] as important mediators of neutrophil antimicrobial function against *B. anthracis*. As a result, we investigated the role of the C3b opsonization of anthrax bacterium and/or PGN in the functional activation of neutrophils. Internalization of bacterial particles was quantified using a pH-sensitive probe, pHrodo Red, whose fluorescence output increases with pH lowering. As such, most of pHrodo fluorescence intensity in live cells is derived from lysosomal particles (pH 4.5) with minimal contribution from surface and early endosomal compartments (pH 7.2). Average internalization of anthrax bacterium and/or PGN within 1 h at 37 °C in the study cohort is shown in Figure 3.

To our surprise, and unlike the binding studies at subphysiologic temperatures above, neutrophils showed a significant uptake of pHrodo- hk*Ba* and/or PGN in the absence of serum factors (mean fluorescence intensity of 5034 ± 195.7 in the absence of PAMPs vs. 12284 ± 3788 for pHrodo- hk*Ba* in the absence of serum, *p* = 0.0012 paired *t* test, and, respectively, 15764 ± 2105 for pHrodo-PGN, *p* = 0.0012 paired *t* test). The uptake was further enhanced by serum opsonization, which was approximately 4-fold for hk*Ba* and doubled for PGN (*p* = 0.0005, and *p* = 0.0009, respectively, compared to paired particle upload in the absence of serum, RM one-way ANOVA). Inhibition of C3b opsonization by compstatin reduced pHrodo-hk*Ba* internalization by 53 ± 21%, (*p* = 0.0016, one sample *t* test, Figure 3A right panel) and reduced pHrodo-PGN internalization by 23%, which approached but did not pass the statistical significance threshold (*p* = 0.0712, one sample *t* test, Figure 3B right panel). None of the complement depleted sera used in the study significantly reduced the internalization of hk*Ba* and/or PGN despite their effect on particle binding at lower temperatures. Our data suggest that C3b opsonization contributes to, but is dispensable for, neutrophil phagocytosis of anthrax bacterium and PGN. Furthermore, they indicate that low-affinity interactions with other immune receptors, likely Fc receptors, drive phagocytosis independent of, or in addition to, complement receptors.

### 3.3. C3b Opsonization Requirements for Neutrophil Degranulation

**Figure 4:**
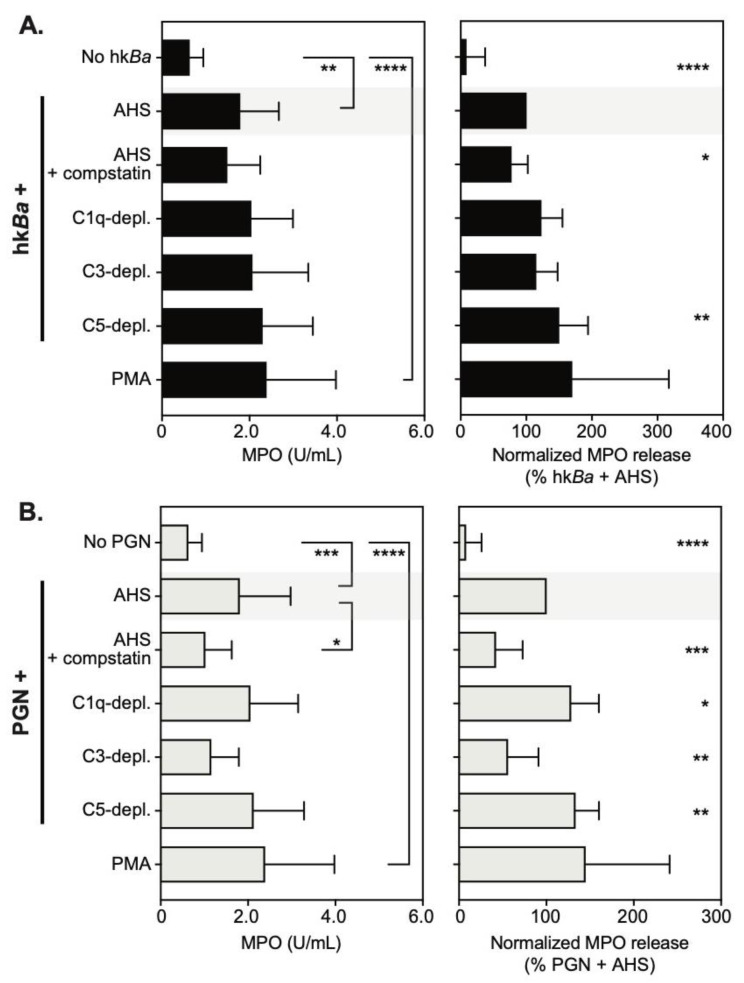
Quantitation of neutrophil degranulation and myeloperoxidase (MPO) release after bacteria (hk*Ba*, **A**) or PGN (**B**) stimulation in the presence of compstatin, a C3 convertase inhibitor, or the immunodepletion of complement factors. Data are shown as mean ± SD of 10 independent donors and depict MPO activity in supernatants collected from stimulated neutrophils (left panels), changes in released MPO after the normalization to bacteria or PGN stimulation in the presence of autologous serum (AHS), which was considered 100% (right panels). Unless otherwise marked, statistically significant differences compared to bacteria and/or PGN stimulation in the presence of AHS (shaded) are depicted graphically (* *p* < 0.05; ** *p* < 0.01; *** *p* < 0.001; **** *p* < 0.0001), and were computed by repeated measures one-way ANOVA with Holm–Sidak’s multiple comparisons test (left panels) or one sample *t* test compared to the normalized value (right panel).

Neutrophils store presynthesized immune mediators in a heterogenous pool of intracellular granules [43], which, upon activation, fuse with either phagosomes and/or the plasma membrane. Myeloperoxidase is an important neutrophil antimicrobial that supports both intracellular and extracellular pathogen killing through oxidizing reactions [44,45]. We thus investigated whether C3b opsonization of anthrax bacterium and/or PGN is required for degranulation and MPO release from neutrophils (Figure 4). We found that both hk*Ba* and anthrax PGN opsonized with autologous serum induced similar release of MPO from activated neutrophils, and the enzyme was active in the extracellular milieu in the presence of hydrogen peroxide (1784 ± 887 mU/mL and 1807 ± 1167 mU/mL, respectively). Anthrax induced MPO release was similar to phorbol-12-myristate-13-acetate (PMA) stimulation (2386 ± 1590 mU/mL), and was significantly higher than cultured neutrophils in the absence of bacterial PAMPs (628 ± 315 mU/mL, *p* = 0.0024 compared to hk*Ba*-stimulated neutrophils, and *p* = 0.0002 compared to PGN-stimulated neutrophils). After pairwise normalization to reduce variability between donors, we observed that compstatin inhibition of the C3b deposition on hk*Ba* reduced MPO release by 23% (*p* = 0.0184, one sample *t* test, Figure 4A). None of the complement immunodepletions investigated reduced MPO release and, surprisingly, C5 depletion in turn enhanced MPO release by 50% (*p* = 0.006). Both compstatin and C3 immunodepletion reduced anthrax PGN by 58% (*p* = 0.0002) and 44% (*p* = 0.0032), respectively (Figure 4B). In contrast, both C1q and C5-immunodepletions enhanced anthrax PGN-induced MPO release. Taken together, we conclude that C3b recognition by complement receptors supports PGN-induced degranulation and MPO release, but is less relevant for hk*Ba*-induced degranulation when other immune receptors, possibly TLRs, could provide redundant signals [32,46].

### 3.4. Complement Enhances Neutrophil MPO Immunoreactivity

**Figure 5:**
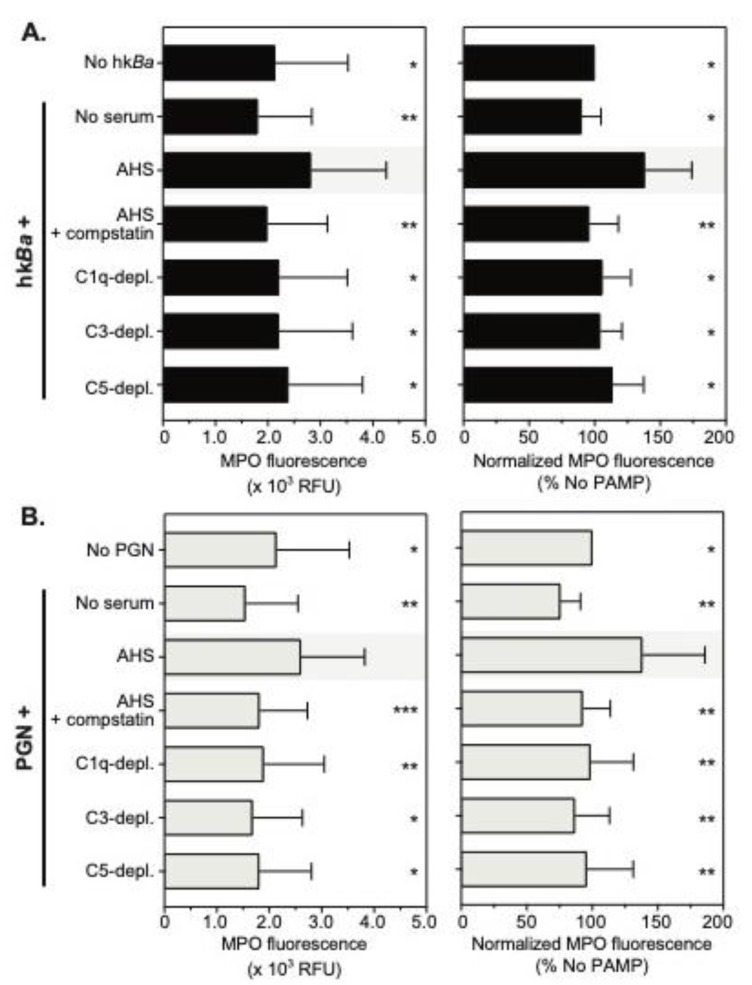
Flow cytometry quantitation of myeloperoxidase (MPO) immunoreactivity in neutrophils stimulated with hk*Ba* (**A**) or PGN (**B**) in the presence of compstatin, a C3 convertase inhibitor, or the immunodepletion of complement factors. Data are shown as mean ± SD of 10 independent donors and the depict intracellular MPO fluorescence (geometric mean) in CD16^high^ neutrophils (left) or the changes in MPO fluorescence after paired normalization to control MPO immunoreactivity in the absence of agonists (right panel). Statistically significant differences compared to bacteria and/or PGN-induced MPO immunoreactivity in the presence of the autologous serum (AHS, shaded) are depicted graphically (* *p* < 0.05; ** *p* < 0.01; *** *p* < 0.001), and were computed by repeated measures one-way ANOVA with Holm–Sidak’s multiple comparisons tests.

PMA is a potent neutrophil agonist that has been shown to promote the release of up to 30% of granular MPO in extracellular milieu, albeit with longer incubation [45]. Since both hk*Ba* and PGN promoted degranulation similar to PMA (Figure 4), we thought to confirm these observations by quantifying residual neutrophil MPO by flow cytometry. Average changes in MPO immunoreactivity, depicted as mean ± SD from 10 independent donors, are shown in Figure 5. Contrary to our expectations, hk*Ba* slightly decreased MPO immunofluorescence compared to unstimulated PMNs in only 2 of the 10 donors studied, by 6% and 7.4%, respectively. In contrast, six of the donors showed a significant upward shift in MPO immunofluorescence of >30%. After pairwise normalization, the average MPO immunofluorescence in the study group increased by 38 ± 36% in response to hk*Ba* opsonized by autologous serum, which was significantly higher than the unstimulated controls (*p* = 0.0160, RM one-way ANOVA, Figure 3A).

Similar to parental bacteria, anthrax PGN decreased neutrophil MPO immunoreactivity in only one donor by 5%, and the MPO fluorescence in two others was virtually unchanged compared to unstimulated controls. The other seven donors showed enhanced MPO immunoreactivity, which varied between 18–160% above the paired controls. After pairwise normalization, the mean ± SD MPO fluorescence in the study group increased by 38 ± 48% in response to PGN opsonized by autologous serum, which was virtually identical to hk*Ba* induced responses and significantly higher than the unstimulated controls (*p* = 0.0324, RM one-way ANOVA, Figure 3B). Stimulation with either hk*Ba* or PGN in the absence of serum, compstatin inhibition, as well as the depletion of any of the three complement factors tested, reduced MPO immunoreactivity to unstimulated control levels (Figure 5). We conclude that both anthrax bacilli and PGN increase MPO immunoreactivity through a serum-dependent mechanism involving complement factor 5, and independent part of C3b recognition. The depletion of upstream factors C1q and C3 attenuate the complement cascade amplification and indirectly reduce C5 processing, resulting in similar outcomes of C5 depletion.

## 4. Discussion

Multiple studies have highlighted an increased role for neutrophils in anthrax pathology and disease progression in rodents [18,19,20,21], and similarly, we observed neutrophil activation in non-human primate models of late stage disease [22,33]. In contrast to their professional phagocyte counterparts [11,12,14,47], the molecular mechanisms responsible for anthrax recognition by neutrophils have not been elucidated. In this study, we investigated the role of C3b opsonization and its recognition by complement receptors for anthrax interaction with neutrophils, and also the subsequent activation of phagocytic and degranulation mechanisms. We found that neutrophils rely on pathogen deposited C3b for recognition of bacteria at suboptimal temperatures. At 37 °C, however, redundant mechanisms reduce their dependence on C3b recognition and, consequently, C3 opsonization supports, but is not critical for, phagocytosis and neutrophil degranulation.

Neutrophils are terminally differentiated immune cells with progressively reduced transcriptional and translational capacity, which exhibit a high reactivity towards pathogens due to presynthesized mediators stored in a heterogenous pool of granules [43]. Neutrophils recognize pathogens through a plethora of immune receptors, including low affinity immunoglobulin receptors FcγRIIA and FcγRIIIB [29]; complement receptors CR1 (CD35), CR3 (CD11b/CD18) and CR4 (CD11c/CD18) [30,31] and the full repertoire of toll-like receptors (TLR), with the exception of TLR3 [32]. Amongst those, FcγRIIA [48] and complement receptors CR1 and CR3 [49] are considered the primary phagocytic receptors on neutrophils, a process modulated by FcγRIIIb engagement [48,50] and/or TLRs [51]. While anthrax bacterium could potentially interact with all the above receptors, anthrax PGN does not bind surface TLR receptors [37], and primarily uses Fc and complement receptors for interaction with myeloid cells [35,39,42,52]. Our current study identifies C3b recognition as a central mediator in neutrophil interaction with anthrax particles, either bacteria or PGN sacculi, at subphysiologic temperatures. Both the inhibition of C3 deposition and/or the immunodepletion of C3 reduced anthrax particles binding by 80–90% under these conditions. These observations are in line with anthrax recognition through CR1/CR3 receptors, which exhibit higher affinities for their cognate C3b ligand [53,54] than the low-affinity immunoglobulin receptors that are constitutively expressed by neutrophils [55]. CR4 is unlikely to contribute to C3b recognition in this setting due to its very low expression on naïve neutrophils in comparison with CR1 or CR3 [56]. In addition, bacteria, but not PGN binding, was reduced by C1q depletion. Although neutrophils express C1q receptors [30], and, furthermore, CR1 can bind multiple complement opsonins including C1q [57], direct recognition of C1q is unlikely to contribute since C3 depletion, where C1q is present at normal circulating levels, is similarly reduced. These results are more likely explained by a preference of complement cascade activation through the classical, immunoglobulin-C1q pathway on the surface of anthrax bacterium. In contrast, we reported previously that purified anthrax PGN initiate complement through the classical and lectin pathways, leading to both C1q- and/or MBL-dependent C3b deposition [42].

In contrast to binding studies that extensively relied on C3 recognition, anthrax internalization at 37 °C was less dependent on C3b-CR interactions. For anthrax bacterium, compstatin inhibition reduced internalization only by half, while C3 depletion reduced internalization by 15%. These suggest that low affinity interactions, which are irrelevant at lower temperatures, support neutrophil activation and phagocytosis at 37 °C. We attribute this internalization to low affinity Fc receptors (FcγRIIIB and/or FcγRIIA), which are more abundantly expressed on neutrophils [29]. Avidity enhances their interaction with multimeric immunoglobulins [58], and likely clustered IgG on the pathogen, and could further shift recognition towards Fc receptors. We have extensively characterized the roles of Fc receptors in anthrax PGN recognition and in the activation of other innate immune cells [35,36,37,38,39,52,59], including FcγRIIA activation on human platelets [42], and we expect similar recognition mechanisms to support neutrophil interactions. Variances in non-complement serum opsonins between autologous and complement depleted sera, such as SAP and/or specific anti-bacterium IgG [39], could then explain the observed quantitative differences between the use of inhibitor and complement depletion. While we cannot exclude at this time TLR contributions to bacterium recognition at 37 °C; these events are not in play for anthrax PGN recognition, which does not interact with either TLR2 or TLR4 [37].

We observed opsonin-independent internalization of anthrax at 37 °C, which accounted for ingestion of 20% bacteria and 40% PGN particles within 1 h. Previous reports recognized that neutrophils kill anthrax bacteria by opsonin-independent mechanisms [23]. Nevertheless, the experimental model was primarily assessing secretion of α defensins and extracellular killing, and, as such, the secretagogue effect of anthrax recognition, and not internalization, which we report here. The quantitative disparities between bacteria and PGN could be reflective of the engagement of different sets of receptors or, alternatively, environmental sampling by neutrophils through macropinocytosis, which is expected to pick up smaller sized PGN more. It is worth noting that CR3 binding to pathogen polysaccharides, like β1,3-linked glucan chains, has been reported and could support opsonin-independent neutrophil activation [60]. While we are unaware of CR3 binding to β1,4-linked glycans, such as anthrax peptidoglycan, β1,3-glucan interaction with CR3 was competitively inhibited by *N*-acetyl-glucosamine, a constitutive PGN sugar [61]. Further studies assessing the putative interaction between anthrax PGN and CR3, or specific inhibition of CR3 in the absence of serum opsonins, are needed to elucidate the role of CR3 in serum-independent anthrax internalization. Altogether, our data indicate that the initial neutrophil ingestion of anthrax bacterium occurs through serum-independent mechanisms (20%), C3b-mediated phagocytosis (40%) and complement-independent low affinity-driven interactions, likely FcγRIIA (40%).

Besides internalization, neutrophil-mediated anthrax killing occurs through the secretion of antimicrobial peptides, like α defensins, in the extracellular milieu [20,23]. Neutrophils store defensins in a subtype of azurophilic granules primarily defined by myeloperoxidase (MPO) storage [43]. Degranulation of azurophilic granules leads primarily to their fusion with, and the subsequent release of, antimicrobial peptides, hydrolytic enzymes and MPO into phagolysosomes, where they assist with intracellular bacterial killing [44]. Azurophilic granules can also exocytose, leading to the release of MPO and defensins, which, in turn, assist with extracellular anthrax killing [20,23]. MPO has been shown to be associated with neutrophil extracellular traps (NETs), and localizes the production of reactive oxygen metabolites in the presence of hydrogen peroxide [45,62]. We found that both anthrax bacterium and PGN acted as secretagogues, promoting azurophilic granule exocytosis and MPO release. C3b opsonization was dispensable for bacterium-induced MPO release. Both compstatin and C3 depletion reduced by half the PGN-dependent MPO release. Since anthrax PGN does not interact with TLR receptors, as we previously reported [37], we interpret these data to indicate that redundancy through TLR signaling promotes exocytosis of azurophilic granules and subsequent MPO and defensin release.

To our surprise, neutrophil degranulation did not corroborate with a reduction of residual neutrophil MPO immunoreactivity, and both the bacterium and anthrax PGN increased MPO immunodetection. At face value, this means more productive antigen (MPO)—antibody interactions that can be attributed to more antigens, or to a better presentation of the antigen during immunostaining. We currently favor the latter. Neutrophils are terminally differentiated leukocytes with reduced, but not eliminated, transcription and translation processes. MPO in particular is primarily synthesized during the early phases of neutrophil development—the myeloblast to myelocyte stages [43]. Although we cannot fully exclude de novo MPO synthesis in response to anthrax recognition, we find the translation of new antigens to account for the enhanced immunoreactivity and MPO exocytosis to be unlikely. Alternatively, azurophilic granules tightly pack multiple immune mediators in highly concentrated vesicles, which might hinder antigen presentation during immunodetection. Priming of azurophilic granules, and/or their fusion with phagolysosomes, is expected to promote a spatial relaxation of the intravesicular content, which could, in turn, allow for more productive antigen-antibody interactions.

Interestingly, the enhanced MPO immunoreactivity was not observed in the absence of serum. This could indicate that the priming of azurophilic granules is independent of phagosome formation since small amounts of bacteria were ingested in the absence of serum opsonization. Alternatively, if serum-independent internalization represents a distinct endocytic pathway, like macropinocytosis, it could, in turn, promote endolysosomal processing without oxidative bursts, similar to processing by dendritic cells. In addition, compstatin, as well as the immunodepletion of every complement factor tested in the study, diminished anthrax and PGN-induced MPO immunoreactivity to levels of unstimulated controls. We conclude that enhanced MPO immunoreactivity, and azurophilic granule priming, relies on proteolytic activation of complement factor C5. We, therefore, hypothesize that complement activation on the anthrax surface, either bacterium and/or PGN, leads to a deposition of C3b opsonin, which helps with pathogen internalization, as well as the release of C5a anaphylatoxin, which, in turn, activates C5aR signaling and the downstream priming of azurophilic granules. Specific modulation of opsonin and receptor signaling, by themselves or in combination, will be required to elucidate these mechanisms. Nevertheless, anthrax bacterium, and/or the purified PGN sacculi of lower structural complexity, could be used to elucidate how neutrophils direct azurophilic granules either towards phagosome fusion and/or plasma membrane fusion and exocytosis, which are cellular events incompletely understood to date.

## 5. Conclusions

Overall, we show that the C3b deposition on anthrax particles is important for affinity recognition by neutrophils; however, C3b-CR interactions support, but are not essential for, either pathogen internalization or neutrophil degranulation at physiologic temperatures. Redundant mechanisms are in play and they involve both opsonin-independent as well as low affinity receptors mediated internalization, likely FcγRIIA. Furthermore, priming of azurophilic granules requires complement C5 activation and co-stimulation of the C5a anaphylatoxin receptor. Although energetically inefficient, maintaining redundant mechanisms for pathogen recognition reduces the risk of microbial evasion and supports the hyperreactivity of these immune sentinels.
